# Genetic dissection of innate immune memory in *Drosophila melanogaster*

**DOI:** 10.3389/fimmu.2022.857707

**Published:** 2022-08-04

**Authors:** Chang Tang, Shoichiro Kurata, Naoyuki Fuse

**Affiliations:** Graduate School of Pharmaceutical Sciences, Tohoku University, Sendai, Japan

**Keywords:** *Drosophila*, *Drosophila* synthetic population resource (DSPR), quantitative trait loci (QTL), *Adgf-A*, innate immune memory

## Abstract

Current studies have demonstrated that innate immunity possesses memory characteristics. Although the molecular mechanisms underlying innate immune memory have been addressed by numerous studies, genetic variations in innate immune memory and the associated genes remain unclear. Here, we explored innate immune memory in 163 lines of *Drosophila melanogaster* from the Drosophila Synthetic Population Resource. In our assay system, prior training with low pathogenic bacteria (*Micrococcus luteus*) increased the survival rate of flies after subsequent challenge with highly pathogenic bacteria (*Staphylococcus aureus*). This positive training effect was observed in most lines, but some lines exhibited negative training effects. Survival rates under training and control conditions were poorly correlated, suggesting that distinct genetic factors regulate training effects and normal immune responses. Subsequent quantitative trait loci analysis suggested that four loci containing 80 genes may be involved in regulating innate immune memory. Among them, *Adgf-A*, which encodes an extracellular adenosine deaminase-related growth factor, was shown to be associated with training effects. Our study findings help to elucidate the genetic architecture of innate immune memory in *Drosophila* and may provide insight for new therapeutic treatments aimed at boosting immunity.

## Introduction

The immune system is traditionally divided into two arms: innate and adaptive immunity. Innate immunity is generally thought to be a primitive immune system since it exists in most multicellular organisms. Innate immunity responds rapidly to a broad range of pathogens as the first line of defense, whereas adaptive immunity functions slowly and later. Adaptive immunity exists only in vertebrates, exhibits high specificity against pathogens, and builds immune memory. Various vaccines have been developed using the memory characteristics of adaptive immunity, saving millions of lives. Innate immunity was not considered to have memory building properties, but an increasing number of studies have suggested the presence of immunological memory in the innate immune response. These memory characteristics have been called “innate immune memory,” “trained immunity,” “immune priming,” “systemic acquired resistance,” and so on (1–4). The broad term “innate immune memory” is used hereafter to encompass these phenomena.

In invertebrates lacking adaptive immunity, prior exposure to pathogen infection (i.e., training) has been shown to protect the host from subsequent infection. For example, prior training with dead *Streptococcus pneumoniae* enhanced the survival rate of flies (*Drosophila melanogaster*) against subsequent challenges with live *S. pneumoniae* (5). A similar phenomenon has been observed with other invertebrates such as mosquitoes, snails, and roundworms (1, 2, 6–8). Moreover, some studies have demonstrated that this immune memory can be transmitted to future generations. A study with shrimp (*Penaeus monodon*) reported that exposing shrimp mothers to β-glucan, which is recognized as a pathogen-associated molecular pattern (PAMPs), resulted in offspring gaining resistance against white spot syndrome-associated virus (9). This type of innate immune memory was denoted as “transgenerational immune priming” (10).

The phenomenon of innate immune memory has also been detected in vertebrates. For example, immunodeficient SCID mice respond to training with PAMPs, exhibiting enhanced survival against subsequent challenges with pathogens (11). Moreover, vaccination with Bacillus Calmette-Guérin (BCG), which protects against tuberculosis, can also improve protection against other pathogens in mice and humans (12–14). The innate immune memory within vertebrates has also been denoted as “trained immunity” (15, 16).

It remains unclear how innate immune memory is conserved among organisms and between vertebrates and invertebrates. Addressing this issue requires resolving the underlying molecular mechanisms in diverse organisms. Current studies with mammals have revealed some of the molecular mechanisms responsible for immune memory. Several studies reported that immune stimulus induced epigenetic reprogramming in innate immune cells and their stem cells (15–17), suggesting that epigenetic reprogramming plays an important role in immune memory. In addition, a metabolic shift in the cholesterol synthesis pathway has been shown to contribute to innate immune memory in mammals (18). Moreover, endoreplication (regional DNA replication without mitosis) was activated during the induction of innate immune memory in vertebrate and invertebrate cells, suggesting its role in modifying gene expression (19). However, many questions remain unanswered, including how epigenetic memory forms during the immune response and how it affects the systemic physiology on individual level.

We previously established an experimental system for detecting innate immune memory in *Drosophila* (Fuse et al., submitted) using *Micrococcus luteus* (Ml) as low pathogenic bacteria for training and *Staphylococcus aureus* (Sa) as highly pathogenic bacteria for subsequent challenge. Prior training with Ml enhanced the survival rate of flies after Sa challenge. Ml bacteria were gradually removed from the fly body after infection, but training effects were sustained after complete removal of Ml. In addition, Ml training suppressed the growth of Sa bacteria in flies after infection, suggesting immune potentiation *via* training. Moreover, the effects induced by Ml training showed a broad range of specificity because fly survival was also significantly increased even after subsequent challenge with *Pseudomonas aeruginosa*. Furthermore, RNA sequencing (RNA-seq) analysis revealed the recall of transcriptional activation after Sa challenge under Ml training conditions (Fuse et al., submitted). To further address the molecular mechanism of innate immune memory, we adopted another approach using genetic variations.

Over the past few decades, genome-wide association studies (GWAS) have facilitated the identification of genes associated with numerous traits and diseases in humans and other organisms (20). GWAS analysis assesses genetic variations in individuals within a population and determines associations with their traits. Quantitative trait loci (QTL) analysis currently enables high-resolution mapping of genetic variations associated with traits (21). These kinds of genome-wide analyses could be applied to *Drosophila* (22, 23), with two population libraries: the Drosophila Synthetic Population Resource (DSPR) (24, 25) and Drosophila Genetics Panel (DGRP) (23, 26, 27). In this study, we performed QTL analysis of innate immune memory using DSPR lines in an effort to dissect the genetic architecture of innate immune memory and identify candidate genes associated with training effects in *Drosophila.*

## Results

### Experimental system for analyzing innate immune memory

**Figure 1:**
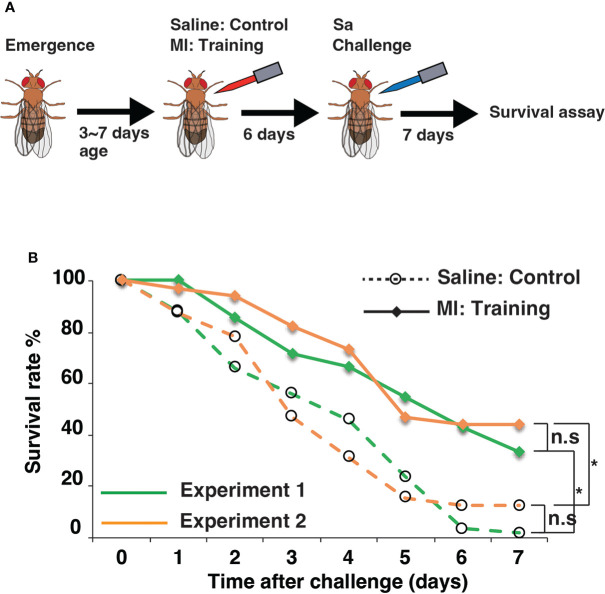
Experimental system of innate immune memory in *Drosophila*. **(A)** Schematic drawing of the systemic infection experiment. Low pathogenic *Micrococcus luteus* (Ml) was injected into the body cavity of flies for training, and lethally pathogenic *Staphylococcus aureus* (Sa) was injected 6 days later for the challenge. Fly survival was monitored for 7 days. **(B)** Survival rates after Sa challenge under control and training conditions. Average survival rates for two experiments are shown in green and orange lines. *, p-value < 0.05; n.s, not significant, determined using the log-rank test and Tukey’s HSD *post-hoc* test. Data of replicates in same experiment are shown in **Supplementary Figure S1**.

Before exploring the genetic basis of innate immune memory in *Drosophila*, we evaluated our experimental assay system (**Figure 1A**). Flies under training conditions were injected with *M. luteus* (Ml) and 6 days later were challenged by injection with *S. aureus* (Sa). Flies under control conditions were injected with saline and were challenged with Sa. The survival rate of flies after Sa challenge was measured for 7 days under training and control conditions (**Figure 1B**). As shown in **Supplementary Figure S1**, survival rates after Sa challenge were similar among replicates under both control and training conditions, suggesting that the survival assay was highly reproducible. Similar increases in survival rates under training conditions were observed between two independent experiments (**Figure 1B**), suggesting that the Ml training effects were also reproducible.

**Figure 2:**
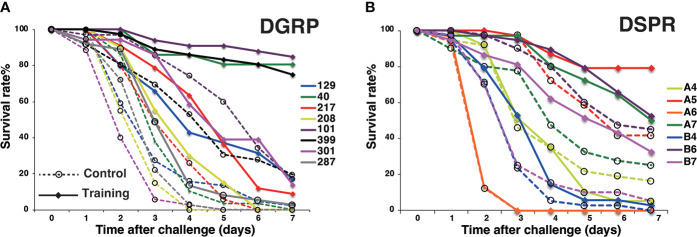
Pilot experiments using the DGRP and DSPR population libraries. **(A)** Survival curves of the eight DGRP lines. DGRP line numbers were shown in the graph. Statistically significant differences between control and training conditions were detected in all lines using the log-rank test. **(B)** Survival curves of seven DSPR founder lines (#A4, #A5, #A6, #A7, #B4, #B6, and #B7). Statistically significant differences between control and training conditions were detected except in lines #A4, #A6, and #B6. Survival curves were measured in two or three independent experiments. Survival rates under control and training conditions are marked by dashed lines and full lines, respectively.

We then performed pilot experiments using *Drosophila* lines from the DGRP and DSPR population libraries. Survival assays were conducted using eight randomly selected DGRP lines and seven founder DSPR lines. Under control conditions, survival rates after Sa challenge varied among DGRP and DSPR lines (**Figure 2**). Under training conditions, survival rates increased among all eight DGRP lines (**Figure 2A**). In the case of the DSPR strains, we detected apparent training effects in four of the seven lines (A5, A7, B4, and B7) but not in the remaining three lines (A4, A6, and B6) (**Figure 2B**). These results indicated that the DSPR lines showed large variations in training effects (including the absence of effects), suggesting the suitability of this population library for the genome-wide analysis of innate immune memory.

### Genetic variations in training effects

**Figure 3:**
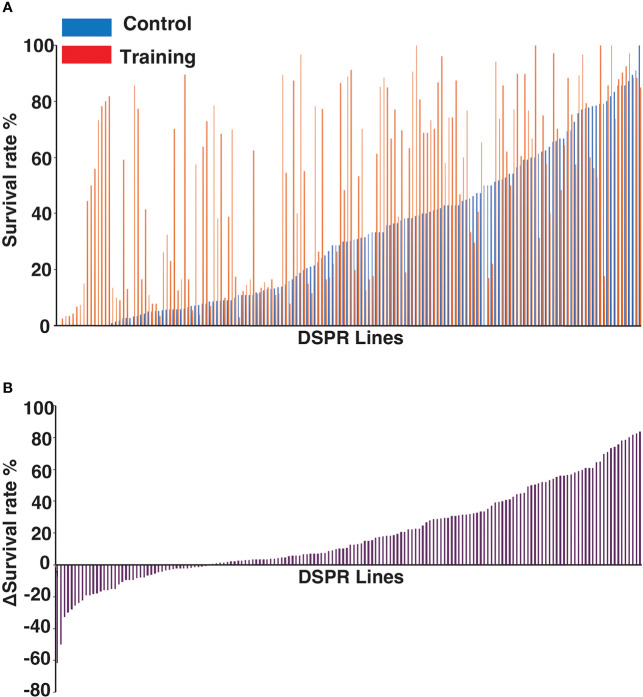
Genetic variations in training effects across DSPR lines. **(A)** Survival rates of 163 DSPR lines on day 3 after challenge under control and training conditions. DSPR lines are ordered according to their survival rates under control conditions. The survival rates of each line are represented by blue (control conditions), and red (training conditions) bars aligned side by side. **(B)** Variations in Δ survival rates (training effects) across 163 DSPR lines on day 3 after Sa challenge. DSPR lines are ordered according to Δ survival rates, which differ from **(A)**. Raw data are shown in **Supplementary Data S1**.

To examine genetic variations associated with innate immune memory, we performed survival assays with 163 *Drosophila* lines from the DSPR population library. The average daily survival rate of each line was monitored for 7 days after Sa challenge under control and training conditions. The survival rates among these DSPR lines varied greatly (**Supplementary Data S1**). For example, the survival rates of 163 lines on day 3 after Sa challenge under control and training conditions were shown in **Figure 3A**. To evaluate the genetic control of survival rates, we calculated the broad-sense heritability of survival on day 3. The heritability of survival was estimated as 63% and 61% under control and training conditions, respectively, indicating that genetic factors significantly contributed to survival under both conditions.

Approximately 72% of the DSPR lines (117/163) survived longer under training conditions than under control conditions (**Figure 3A**), suggesting that training positively affected the survival of most lines. However, the survival rates of five lines were unaffected by training and the remaining 41 lines (approximately 25%) showed decreased survival rates under training conditions compared with control conditions. In such cases, the Δ survival rates were negative (**Figure 3B**). We consider that negative effect is also one of the outcomes of immune training (see Discussion). Thus, the Δ survival rates ranged from -63 to +81% among DSPR lines on day 3.

The Δ survival rates were also calculated at other time points after Sa challenge. As shown in **Supplementary Figure S2**, the Δ survival rates varied from -60 to +90% on days 2 to 6 after Sa challenge (**Supplementary Data S1**). The data of day 1 and day 7 was dismissed from the following analysis, since the survival rates of most strains were nearly 100% on day 1 and almost 0% on day 7.

### Poor correlation between training effect and basal immune response

**Figure 4:**
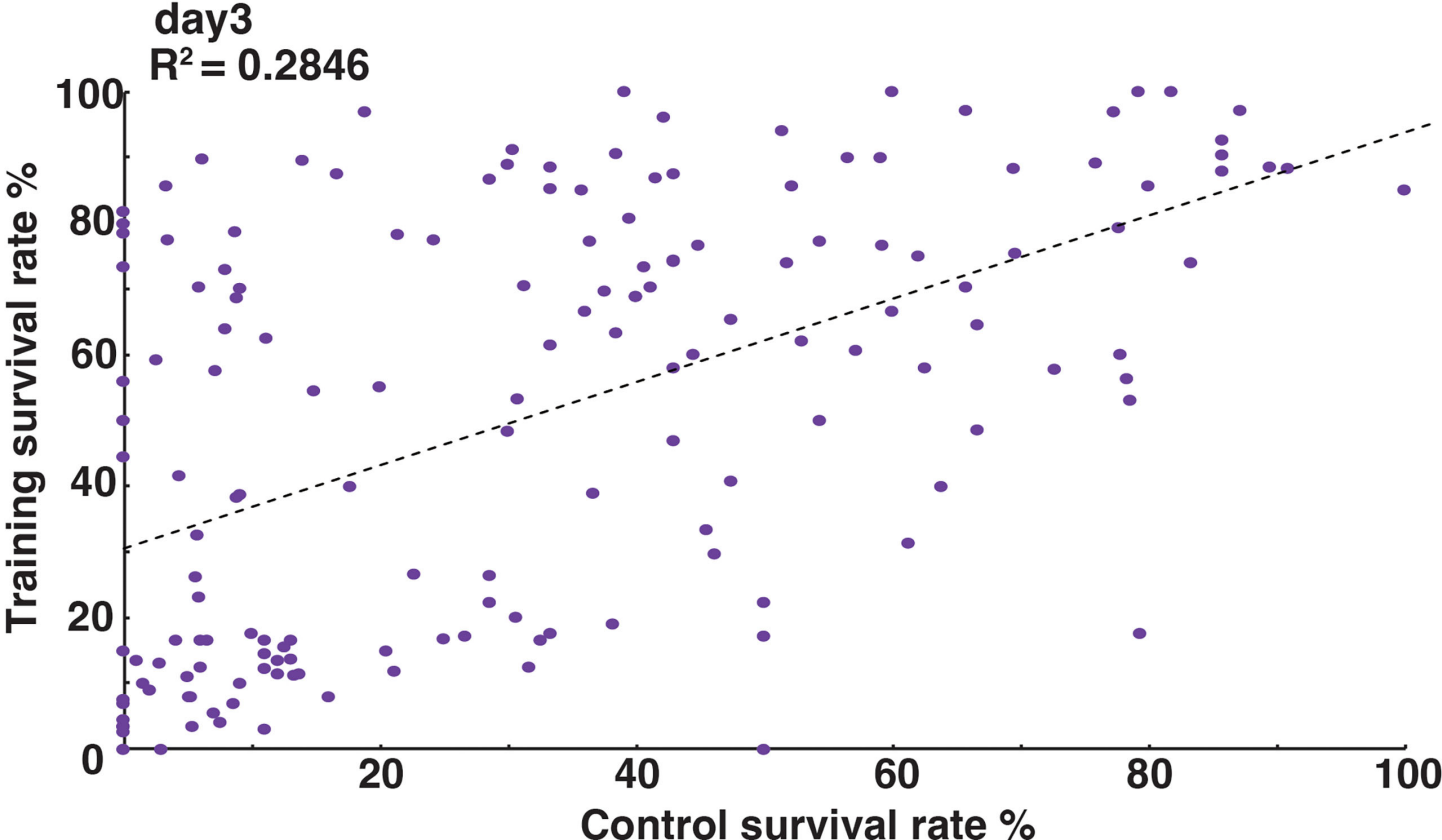
Relationship between normal immune responses and training effects. Scatterplot of survival rates on day 3 after Sa challenge under control (x-axis) and training (y-axis) conditions. Each dot represents a single DSPR line. Survival rates under control and training conditions were poorly correlated (R^2^ = 0.2846). Scatterplots for other time points are shown in **Supplementary Figure S4**.

The order of survival rates between two *Drosophila* lines was often reversed under control and training conditions. For example (**Figure 2B**), the survival rate of line #B7 (purple, dashed line) was lower than that of line #A4 (yellow, dashed line) under control conditions, but was higher than that of line #A4 under training conditions (purple and yellow full lines). Therefore, we compared survival rates under control and training conditions across 163 DSPR lines. As shown in **Figure 4** (survival rates on day 3 after challenge), we found that there was a poor correlation between the survival rates under control and training conditions (correlation coefficient: R^2^ = 0.28), though the statistical test indicated significant correlation between them (p-value = 2.6e-13). Similarly, poor correlations were observed at other time points after Sa challenge (**Supplementary Figure S3**). These results suggested that the genetic controls of training effects are largely different from that of normal immune responses, even though they are not completely independent.

**Figure 5:**
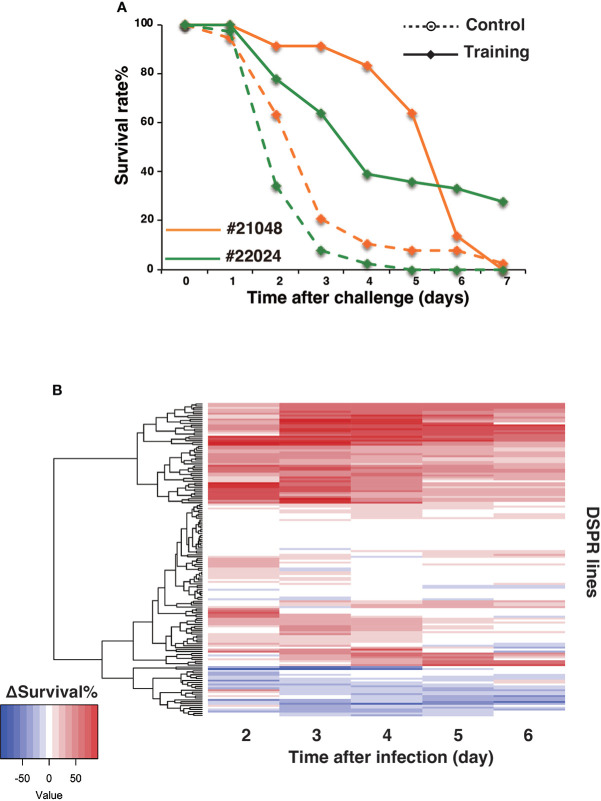
Variations in survival curves and Δ survival rates. **(A)** Data from DSPR lines #21048 (orange) and #22024 (green), representative of variations in survival curves. Survival rates under control and training conditions are marked by dashed and full lines, respectively. **(B)** Heatmap of Δ survival rates of 163 DSPR lines on days 2 to 6 after Sa challenge. 163 DSPR lines (rows) were aligned by the dendrogram of clustering analysis of Δ survival rates.

The shapes of the survival curves often varied for DSPR lines under control and training conditions. For example, the survival curves of DSPR lines #21048 and #22024 were similar under control conditions (**Figure 5A**) but exhibited different shapes under training conditions. Consequently, the Δ survival rates over time showed different patterns for these two lines. A heatmap was used to analyze the Δ survival rate patterns of all 163 lines on days 2 to 6 after Sa challenge (**Figure 5B**). Approximately half of the time windows for all DSPR lines (from days 2 to 6) showed positive training effects (red color), but the temporal patterns of Δ survival rates differed among lines. Namely, some lines maintained constant Δ survival rates during this period, whereas some lines showed early or late peaks in the Δ survival rate.

### Genome-wide QTL analysis of innate immune memory

To identify genetic loci associated with innate immune memory, we initially analyzed loci related to normal immune responses, i.e. using survival rates only under control conditions. Logarithm of the odds (LOD) scores were calculated at 10 kb intervals along the genome on day 3 after Sa challenge, revealing three LOD peaks (**Figure S4**). These results suggested that genes located in these three loci might be responsible for genetic variations in survival after Sa infection and thus for normal immune responses. Among the three detected LOD peaks, one peak on the X chromosome (position 21,990,000) overlapped with one of five QTLs previously associated with immunity against virus infection in *Drosophila* (28). This remarkable overlap suggested that this locus might be involved in normal immunity against bacteria and viruses, thus supporting the validity of our approach.

**Figure 6:**
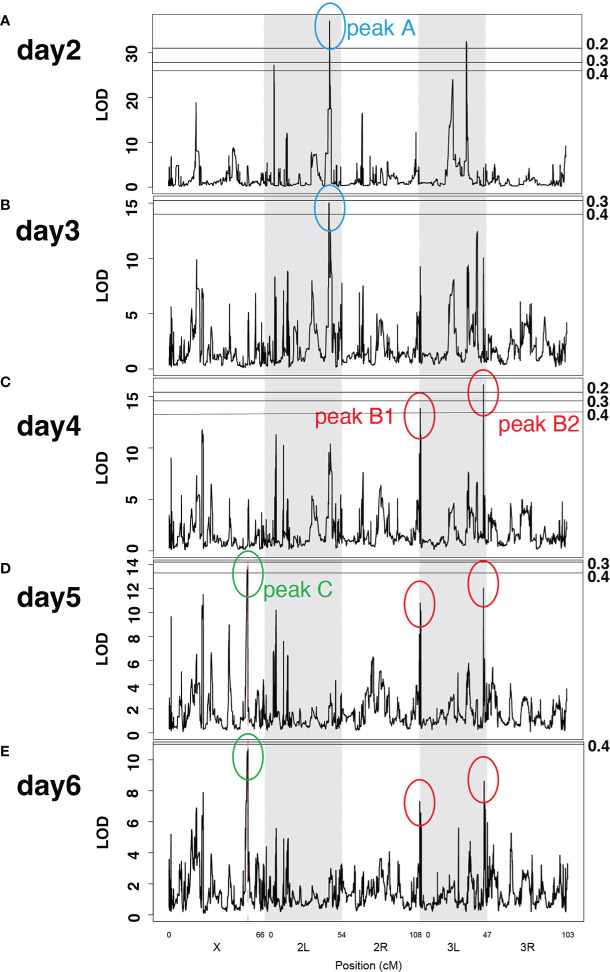
LOD peaks of training effects at different time points after challenge. Line graphs of LOD scores across *Drosophila* main chromosomes (X, 2L, 2R, 3L, 3R) on days 2–6 **(A–E)** after Sa challenge. Peak A was detected on days 2 and 3; peaks B1 and B2 were detected on days 4–6; peak C was detected on days 5 and 6. The X-axis represents the position of chromosomes. The Y-axis shows the LOD scores for each genomic position. Transverse lines represent the LOD scores corresponding to each false positive rate (numbers labeled on the right). Information on LOD peaks is provided in **Table 1**.

Next, we searched for genetic loci associated with training effects. Using normalized hazard ratios (29, 30), we calculated LOD scores of training effects along the genome on days 2–6 after Sa challenge (**Figure 6**). We detected several LOD peaks along the genome, among which the four highest peaks were commonly detected at several time points after Sa challenge. As shown in **Figures 6A, B**, peak A was detected on both days 2 and 3 after Sa challenge. Similarly, peaks B1 and B2 were both detected on days 4–6 (**Figures 6C–E**), and peak C was detected on days 5 and 6 (**Figures 6D, E**). Although LOD score values varied between time points, the region and shape of the LOD peaks were conserved (**Supplementary Figure S5**). To estimate the statistical threshold of LOD scores, we performed a 1000-permutation test and calculated the genome-wide false-positive rate. Based on previous studies (25, 27), the false positive rate was set at 0.5 (50% chance of a single false-positive result). All four detected LOD peaks exceeded this criterion (false-positive rate < 0.5) (transverse lines in **Figure 6**). Furthermore, the reproducibility of the LOD peaks were validated in randomly selected 123, 143, and 157 lines from the total 163 DSPR lines. Although LOD score values varied between these data sets, most LOD peaks were conserved (**Supplementary Figure S6**). These results indicated that the four LOD peaks would be stably detected irrespective of the data set, suggesting that these genetic loci would be relevant to training effects. Indeed, QTL analysis estimated that these LOD peaks contributed to 32–65% of the variation in the training effects (**Table 1**). Consequently, we detected the four LOD peaks at different time points after Sa challenge (**Figure 6**): peak A on days 2 and 3, peaks B1 and B2 on days 4–6, and peak C on days 5 and 6.

**Table 1:** QTL/LOD peaks identified in QTL analysis.

QTL/Peak #	Chr	Highest position (kb)	Highest LOD score	Percent variation	Peak left (kb)	Peak right (kb)	Range	Genes included
A	2L	12110	37.0	65.2	12110	12390	280kb	21
B1	3L	1290	13.8	32.5	1280	1310	30kb	7
B2	3L	17790	16.2	36.8	17730	17800	70kb	15
C	X	16590	13.8	32.5	16340	16590	250kb	37

For each QTL/LOD peak, peak name, chromosome, position, LOD score, contribution rate % to variation, peak range, and number of the included genes are indicated. Genome positions are based on release 5 of the D. melanogaster genome sequence (BDGP R5/dm3). Name of the included genes are shown in **Supplementary Table S1**.

To identify candidate genes involved in innate immune memory, we defined the genome positions of these LOD peaks according to their shape (**Supplementary Figures S7–S10**). The genes located within the four LOD peaks (peaks A, B1, B2, and C; **Table 1**) were identified, revealing 80 candidate genes that could be involved in innate immune memory (**Supplementary Table S1**). These candidate genes included some genes related to the immune response (*Adgf-A*, *Ubc7*, *Rac1*, and *NUCB1*) and epigenetic gene regulation (*scf*, *Ctr9*, *SMC3*, and *Nup153*).

### Involvement of *Adgf-A* in training effect

To narrow down the candidate genes involved in innate immune memory, we utilized data from our previous RNA-seq analysis in which we examined gene expression changes after Ml training and Sa challenge (Fuse et al., submitted). Integrative analysis indicated that eight genes were shared between the candidate genes of the QTL analysis and the differentially expressed genes (DEGs) of the RNA-seq analysis, including *Acat2, Adgf-A, atilla*, lncRNA *CR44668, Phae1, Phae2, scf*, and *ZnT33D* genes.

**Figure 7:**
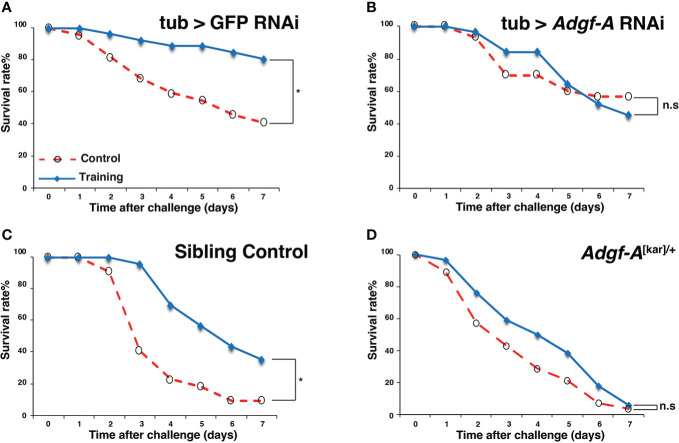
Involvement of *Adgf-A* in training effects. Survival curves of flies subjected to Ml training and Sa challenge. Blue and red lines represent survival rates under training and control conditions, respectively. **(A)** Control RNAi line (tub > GFP RNAi, training with Ml, OD = 2), **(B)** *Adgf-A* RNAi line (tub > *Adgf-A* RNAi, training with Ml, OD = 2), and **(C)** Sibling control (*Adgf-A* ^+/+^) of **(D)**, **(D)** *Adgf-A* mutant heterozygote (*Adgf-A* ^[kar]/+^). *, p < 0.05; n.s., not significant, determined using the log-rank test. The numbers of flies used in these experiments were **(A)** 22, 26, **(B)** 30, 31 **(C)** 22, 23, **(D)** 28, 34 (under training or control conditions, respectively).

In this study, we focused on one candidate gene, *Adgf-A* (FBgn0036752), which encodes an adenosine deaminase-related growth factor associated with the immune response (31). Survival assays were performed using *Adgf-A* RNAi line (*UAS-Adgf-A* RNAi) (32) and *Adgf-A* mutant line (*Adgf-A* ^[kar]^) (33). As shown in **Figures 7A, B**, the control RNAi line (tub > GFP RNAi) exhibited increased survival after Sa-challenge under the Ml-training condition, but the *Adgf-A* RNAi line (noted as tub > *Adgf-A* RNAi) showed no training effect. Moreover, *Adgf-A* mutant heterozygote (noted as *Adgf-A* ^[kar]/+^) abolished the effect of Ml-training (**Figure 7D**), while the sibling control line (*Adgf-A* ^+/+^) showed apparent training effect (**Figure 7C**). These results supported that *Adgf-A* plays an essential role in inducing training effects on survival.

**Figure 8:**
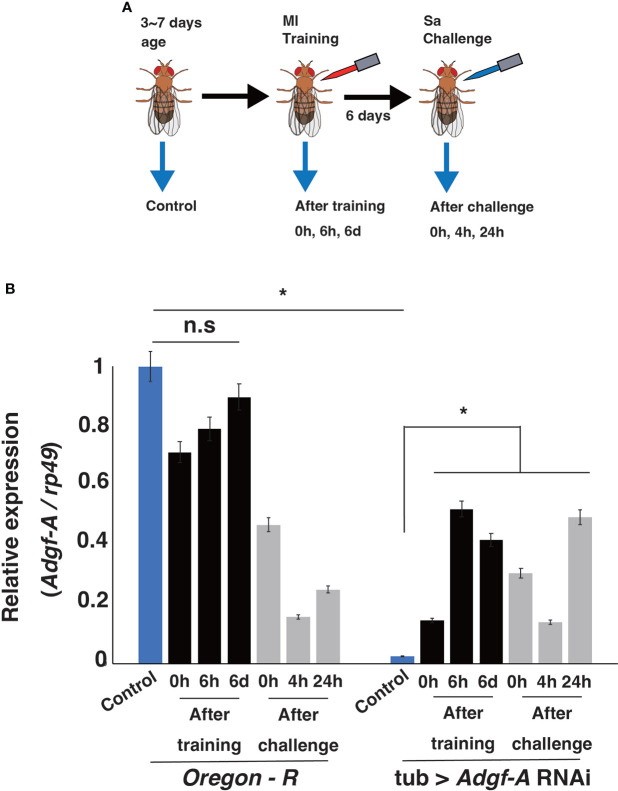
*Adgf-A* expression after training and challenge. **(A)** Experimental design for RT-qPCR analysis. Adult flies from 3 ~ 7 days old (blue bars) and the flies at 0 h, 6 h, and 6 d after Ml training (black bars) were sampled. Moreover, 6 days after Ml training, flies were sampled at 0 h, 4 h, and 24 h after Sa challenge (gray bars). **(B)** RT-qPCR analysis of relative expression of *Adgf-A* against that of *rp49* under each condition. *, p < 0.05; n.s., not significant, determined using one-way ANOVA.

Our previous RNA-seq analysis indicated that *Adgf-A* expression was downregulated after Sa challenge (Fuse et al., submitted). Therefore, RT-qPCR analysis was conducted to measure *Adgf-A* expression in wild-type and knockdown lines in the current study (**Figure 8**). In the wild-type line, *Adgf-A* expression decreased quickly (within 5 min) after Ml training, recovered over 6 days, and was significantly suppressed by Sa challenge. In the *Adgf-A* RNAi line, *Adgf-A* expression was extremely low before Ml training but unexpectedly increased after Ml training. However, its expression level was still low compared with the wild-type line. These results indicated that *Adgf-A* expression was dynamically changed by Ml training and Sa challenge, and such regulation was disturbed in the RNAi line. We speculate that the fine-tuned expression of Adgf-A might contribute to the training effect.

Taken together, our genome-wide QTL analysis identified candidate genes potentially associated with innate immune memory, among which *Adgf-A* was shown to be involved in training effects.

## Discussion

In this study, we first examined variations in training effects among some *Drosophila* lines from the DGRP and DSPR population libraries, which are commonly used for genome-wide population analyses such as GWAS and QTL analyses. In our pilot experiment, the DSPR lines showed larger variations in training effects (including the absence of effects) than the DGRP lines (**Figure 2**). The DGRP library consists of wild-type lines collected in one city in the USA (23), whereas the DSPR library contains a genetic admixture of founder lines collected from different areas around the world (22). Therefore, it is possible that large variations in training effects among the DSPR lines might be attributed to genetic variations selected under diverse environments around the world.

Subsequently, we performed survival assays under training and control conditions using 163 DSPR lines. Many lines (~ 72%) showed positive training effects, while some lines (~25%) showed negative training effects (**Figure 3**). Both the positive and negative effects on survival may have been the outcomes of immune training. In the case of negative effects, dysregulation of the immune response after training may have become detrimental, causing effects such as sepsis. Alternatively, immune signaling may have become non-responsive after training, causing immune suppression. The causal factors responsible for opposite training effects may be revealed through further genetic study.

Survival rates under control and training conditions among the 163 DSPR lines were poorly correlated (**Figure 4**), although statistical analysis indicated significant correlation between them. This finding indicates that survival rates under control and training conditions were not completely independent, but were largely different, suggesting that the mechanisms underlying immune training effects differ from those underlying normal immune responses.

The QTL analysis for training effects revealed four predominant LOD peaks at different time points after Sa challenge. Peak A was detected on days 2 and 3, peaks B1 and B2 were detected on days 4-6, and peak C was detected on days 5 and 6 (**Figure 6**). These results suggested that different loci (genes) might be involved in training effects during different time periods. This idea was consistent with diverse patterns of survival rates under training conditions (**Figure 5**). The survival pattern represents disease progression and is defined by multilayered mechanisms, including immunity, physiology, and metabolism. Hence, we speculate that training effects might also be controlled by multilayered mechanisms involving many genes.

A total of 80 candidate genes were identified with potential involvement in innate immune memory in *Drosophila*, and eight of them were differentially expressed in response to training and/or challenge in the RNA-seq data obtained in our previous experiments. Among them, we focused on *Adgf-A* because this gene is known to be involved in the immune response in *Drosophila* (32). We then examined the effects of *Adgf-A* loss of function on innate immune memory. We found that training effects were impaired in both RNAi and heterozygous mutant lines (**Figure 7**), suggesting that *Adgf-A* contributes to innate immune memory, as well as to normal immune responses. Additionally, *Adgf-A* expression rapidly decreased in wild-type flies after Ml training, gradually recovered, and then was strongly suppressed by Sa challenge (**Figure 8**). Controversially, a previous study reported that *Adgf-A* was upregulated in *Drosophila* after infection with *Listeria monocytogenes* and *Streptococcus pneumoniae* (32). Taken together, these data suggest that *Adgf-A* expression is dynamically regulated during the immune response in a complex manner, which may depend on both infection conditions and the bacterial species involved. Thus, we speculate that finely tuned *Adgf-A* expression may contribute to innate immune memory.

*Adgf-A* encodes adenosine deaminase-related growth-factor A and is homologous to the human *ADA2* (*adenosine deaminase 2*) gene. Briefly, ADA2 protein is secreted from myeloid lineage cells and converts adenosine into inosine, thereby regulating the level of extracellular adenosine (32). Adenosine is a signaling molecule that plays important roles in metabolism and immunity (34). Mutations in the human *ADA2* gene cause autoinflammatory diseases, such as deficiency of adenosine deaminase 2 (DADA2), which is characterized by recurrent fever, livedoid rash, stoke, immunodeficiency, and bone marrow failure (35, 36). Previous studies have shown that ADA2 regulates the proliferation and differentiation of monocytes and macrophages *via* regulation of adenosine levels (37, 38). Similarly, *Adgf-A* in *Drosophila* regulates the differentiation of hemocytes (fly macrophages) and balance of energy consumption between immunity and metabolism (32, 39). Moreover, both *Adgf-A* and ADA2 possess growth-factor activities without the involvement of adenosine under some conditions (31).

Although the study findings suggest a previously unidentified role for *Adgf-A* in innate immune memory, detailed mechanism remains unclear. Additionally, the functions of *Adgf-A* in the normal immune response compared to those in immune memory warrant further study. Also, whether *Adgf-A* signals through adenosine for immune memory needs to be elucidated. Notably, the DSPR lines carry many single nucleotide polymorphisms (SNPs) on coding regions and surrounding regions of *Adgf-A* (**Figure S11**), which may be responsible for the genetic variations in training effects displayed among DSPR lines. The effects of SNPs on the expression of *Adgf-A* or the activity of Adgf-A protein remain unclear. These are important issues to be solved in future research.

In conclusion, this study investigated the genetic factors responsible for innate immune memory through a genome-wide QTL analysis of *Drosophila* lines. The study findings indicate that genetic control of innate immune memory is largely different from that of the normal immune response, with some genetic loci contributing to training effects in a complex manner. A total of 80 candidate genes were identified that may be associated with immune training effects in *Drosophila*, among which the involvement of *Adgf-A* was explored. Other identified genes may also be involved in innate immune memory, thus further analyses are needed to clarify their roles. The study findings provide insights that may lead to the development of new therapeutic approaches for boosting immunity.

## Materials and methods

### Flies

*Drosophila* lines from the DSPR population library were utilized in this study. The DSPR is composed of two libraries (A and B) of recombinant inbred lines (RILs), each created by intercrossing a different set of eight founder lines (22). We randomly chose 163 lines from the B library in this study (**Supplementary Data S1**). The DSPR lines were provided by Dr. Stuart J. Macdonald, University of Kansas. The DGRP lines were provided by the Bloomington Drosophila Stock Center (BDSC). #28141 (DGRP-129), #29651 (DGRP-40), #28154 (DGRP-217), #25174 (DGRP-208), #28138 (DGRP-101), #25192 (DGRP-399), #25175 (DGRP-301), and #28165 (DGRP-287) were used. To induce *Adgf-A* RNAi, we crossed tub-Gal4 driver flies (BDSC #5138) and UAS-*Adgf-A* RNAi flies (BDSC #67233), and their offspring (tub > *Adgf-A* RNAi) were used for experiments. As a control, we crossed the tub-Gal4 and UAS-GFP RNAi lines (BDSC #9330), and their offspring (tub > GFP RNAi) were used for experiments. The *Adgf-A* ^[kar]^ mutant line was a gift from Dr. Tomas Dolezal, University of South Bohemia (32). This mutant was created by homologous recombination and was identified as a loss-of-function mutant (33). The mutant line was crossed with w^1118^ (BDSC #3605), and their offspring (*Adgf-A* ^[kar]/+^ and sibling control *Adgf-A* ^+/+^) were used for the survival assay. Flies were reared using the standard cornmeal medium in a 30 mL vial at 25°C in an incubator.

### Bacteria

Two types of bacteria were used in this study. *Micrococcus luteus* (IFO13867) termed as Ml was used for training, and *Staphylococcus aureus* (ATCC10801) and (Xen29) (40, 41) (gift from Chikara Kaito at Okayama University) termed as Sa was used for challenge infection. Ml was cultured in LB medium (Nacalai Tesque, Kyoto, Japan) at 30°C. Sa was cultured in tryptic soy broth (TSB) medium (Becton, Dickinson and Company, USA) supplemented with kanamycin (200 μg/mL) at 37°C because Sa carries the kanamycin-resistance gene.

Ml bacteria were cultured overnight (~16 h) and precipitated by microcentrifugation at 12000 rpm for 10 min at 4°C. The bacterial pellet was resuspended in saline and the concentration was adjusted to OD = 1 for injection (except for tub > GFP RNAi and tub > *Adgf-A* RNAi, which received Ml at OD = 2, in **Figures 7A, B**). Sa bacteria were re-cultured for 3 h at a 1/100 dilution of the overnight culture. Sa bacteria were precipitated and diluted to OD = 0.01 for injection [Xen29, except **Figures 1** and **2**, which received Sa (ATCC10801) at OD = 0.1].

### Survival assay

Adult male flies aged 3–7 days after emergence were used for the survival assay (**Figure 1**). Bacterial solution or saline (control) was filled into a glass needle (3 1/2 inch capillary; Drummond Scientific, Philadelphia, PA, USA), and 69 nL of the solution was injected into the body cavity of the fly thorax using a micromanipulator (Nanoject II; Drummond Scientific). Challenge injection was performed 6 days after training. Flies that died within 18h of Sa injection, independently of experimental conditions, were omitted from counting for the survival assay. For QTL analysis, two vials containing approximately 20 flies per vial were set for each DSPR line and condition (with or without training), and the mean survival rates of the two vials were calculated. The Δ survival rate was calculated by subtracting the survival rate under the control condition from that under the training condition.

Statistical comparison of survival curves was performed using the log-rank test and *post-hoc* Tukey HSD test in R software. The survival rates of each line on each day after Sa challenge are shown in **Supplementary Data S1**.

The broad sense heritability value represents the contribution of genetic factors to an organism’s trait. According to the method described by the DSPR project (http://wfitch.bio.uci.edu/~dspr/index.html) (42), the estimated genetic variance component over the total variance of data was calculated from the survival data using the “lme” and “VarCorr” functions of the “nlme” package in R software.

The correlation coefficient between survival rates under control and training conditions was calculated at each time point after Sa challenge using Microsoft Excel. The statistical correlation test was performed using Pearson’s correlation method with the “corr.test” function in R software.

### Data analysis for QTL

The quotient of survival rates under control and training conditions for each DSPR line (**Supplementary Data S1**) was calculated as a hazard ratio (HR) and normalized as previously described (29, 30). Normalized HRs were used for the QTL analysis, which was performed using the “DSPRqtl” package in R software as described by the DSPR project (http://wfitch.bio.uci.edu/~dspr/Tools/index.html) (42). The *Drosophila* genome was provided in the “DSPRqtlDataB” analysis package. Phenotype data were generated with DSPR RILs as described by the “DSPRqtl” package manual. The “DSPRscan” function was used to perform a genome scan of the generated phenotype data. Through this genome scan, the relationship between HRs and genomic loci was calculated, represented as LOD scores. QTL peaks were extracted using the “DSPRpeaks” function, which located and summarized QTL peaks. The threshold of each QTL peak at different p-values was analyzed using the “DSPRperm” function, which performed a 1000-permutation test for each DSPR dataset. Based on previous studies (25, 27), the genome wide false positive rate was set at 0.5 (50% chance of a single false-positive result). LOD graphs were created using the “DSPRplot” function, which plotted the genome scan results for the DSPR RILs. The four highest LOD peaks were analyzed using the LOD score data from the genome scan. The genes within these four LOD peaks were identified using the UCSC Genome Browser on D. melanogaster Apr. 2006 (BDGP R5/dm3). All QTL data within this paper are based on release 5 of the *D. melanogaster* genome sequence.

### RT-qPCR analysis

Whole adult flies were homogenized, and total RNA was extracted using TriZOL reagent (Invitrogen, Carlsbad, CA, USA). Each sample contained three adults, and three samples were analyzed as biological replicates for each condition. cDNA was synthesized from the total RNA using ReverTra Ace (Toyobo, Osaka, Japan) according to the manufacturer’s protocol. The qPCR reaction was carried out on the Light Cycler 96 (Roche, Basel, Switzerland) using FastStart DNA Master SYBR Green reagent (Roche). The expression of *Adgf-A* was analyzed using the ΔCt method and normalized to that of ribosomal protein 49 (*rp49*). The relative expression (fold change) of *Adgf-A* was compared to that in uninfected Oregon-R flies. The primers for *rp49* were as follows: Fwd 5’-AGATCGTGAAGAAGCGCACCAAG-3’ and Rev 5’-CACCAGGAACTTCTTGAATCCGG-3’. The primers for *Adgf-A* were as follows: Fwd 5’-ATGTCATATAGCGTGGGAAC-3’ and Rev 5’- ATGTGCGAGCCAAATACGG-3’ (32). Statistical comparison of relative gene expression was performed using one-way ANOVA in R software.

## Data availability statement

The datasets presented in this study can be found in online repositories. The names of the repository/repositories and accession number(s) can be found in the article/**Supplementary Material**.

## Author contributions

CT performed experiments. NF and CT designed the experiments and analyzed the data. NF and SK supervised the study. All authors obtained the grants, discussed the results, and wrote the manuscript. All authors contributed to the article and approved the submitted version.

## Funding

This work was supported by a Sasakawa Scientific Research Grant from the Japan Science Society (JST SPRING grant number JPMJSP2114), the System Design of Inclusive Society with Infectious Diseases (SDGS-ID) of Tohoku University, and the Japan Society of the Promotion of Science (JSPS KAKENHI grant numbers 17K07239 and 19H03365).

## Acknowledgments

We especially thank Dr. Stuart J. Macdonald (University of Kansas) for providing the DSPR lines and technical advice. We appreciate Dr. Chikara Kaito (Okayama University) for providing the Sa bacterial strain, Dr. Tomas Dolezal (University of South Bohemia) for providing the *Adgf-A* ^[kar]^ heterozygous mutant line, and the Bloomington Drosophila Stock Center (BDSC) for providing various transgenic fly stocks. We also thank the members of the Kurata Laboratory for their discussion and suggestions.

## Conflict of interest

The authors declare that the research was conducted in the absence of any commercial or financial relationships that could be construed as a potential conflict of interest.

## Publisher’s note

All claims expressed in this article are solely those of the authors and do not necessarily represent those of their affiliated organizations, or those of the publisher, the editors and the reviewers. Any product that may be evaluated in this article, or claim that may be made by its manufacturer, is not guaranteed or endorsed by the publisher.

## Supplementary material

Supplementary Figure 1 Reproducibility of survival rates in our assay system. **(A)** Survival rates of wild-type flies (Oregon-R) after Sa challenge under control conditions. **(B)** Survival rates of Oregon-R after Sa challenge under training conditions. The survival assay was performed in two independent experiments consisting of five biological replicates in total. Differences among five replicates in **(A)** and **(B)** were not statistically significant (log-rank test). The numbers of flies used in these experiments were **(A)** 91 (control) and **(B)** 76 (training).

Supplementary Figure 2 Variations in training effectsVariations in Δ survival rates across 163 DSPR lines on **(A)** day 2, **(B)** day 4, **(C)** day 5, and **(D)** day 6 after Sa challenge. DSPR lines are ordered according to Δ survival rates. Raw data are shown in **Supplementary Data S1**

Supplementary Figure 3 Scatterplots of control and training survival rates. Scatterplots of survival rates under control (x-axis) and training (y-axis) conditions on **(A)** day 2, **(B)** day 4, **(C)** day 5, and **(D)** day 6 after Sa challenge. Each dot represents a single DSPR line. Survival rates under control and training conditions were poorly correlated. Although the correlation coefficient was low, statistical analysis indicated the survival rates were significantly correlated between conditions (p < 0.05).

Supplementary Figure 4 LOD peaks related to normal immune responses. LOD graph of normal immune responses calculated by survival rates under control conditions on day 3 after Sa infection. The highest three LOD peaks (X; 21,990,000, 2R; 9,140,000, and 3R; 21,620,000) are marked in the figure. QTL1 on X chromosome (21,990,000 position) overlapped with one of the five QTLs previously associated with virus immunity in *Drosophila* (see text).

Supplementary Figure 5 Comparison of the shapes of LOD peak on different days. Line graphs of LOD scores for peak B1 on **(A)** day 4 and **(B)** day 6 after Sa challenge. The shapes of these peaks along the local position (3L: 1260000_1560000) were extremely similar.

Supplementary Figure 6 Stable detection of main LOD peaks. LOD scores were calculated from randomly selected DSPR lines. Data of **(A)** 123, **(B)** 140, **(C)** 157, and **(D)** all 163 DSPR lines on day 3 after Sa challenge were used. Prominent LOD peaks, including peak A, were conserved in these analyses.

Supplementary Figure 7–S10 LOD peaks and gene structures. Detailed shapes of peak A (on day 2 and 3), peaks B1 (on day 4-6), peak B2 (on day 4-6), and peak C (on day 5 and 6). Gene structures on the corresponding genomic regions were obtained from the University of California Santa Cruz (UCSC) genome browser (BDGP R5/dm3). See also **Supplementary Table 1**.

Supplementary Figure 11 Genome view around *Adgf-A* gene. The genome view of approximately 14 kb region on 3L chromosome around *Adgf-A* was obtained from UCSC Genome Browser (BDGP R5/dm3). SNPs carried by DSPR lines are shown. Upper and lower nucleotides are referenced and alternate alleles on each position are marked.

Supplementary Table 1 List of candidate genes. A list of 80 candidate genes identified in QTL analysis. The LOD peak, gene symbol, Flybase ID, GO terms (biological process, cellular component, molecular function), location arm, location max, location min, and gene name are listed.

Supplementary Data Sheet 1 Raw data of survival rates of each DSPR line. DSPR line, survival rates under control conditions, survival rates under training conditions, Δ survival rate, hazard ratio (HR), and normalized HR are listed at each time point after Sa challenge.
