# Supplementary figures and images for: Genetic dissection of innate immune memory in Drosophila melanogaster
Source: Front Immunol. 2022 Aug 4;13:857707. doi: 10.3389/fimmu.2022.857707 (PMC9386478; doi:10.3389/fimmu.2022.857707)

# Fig. S1

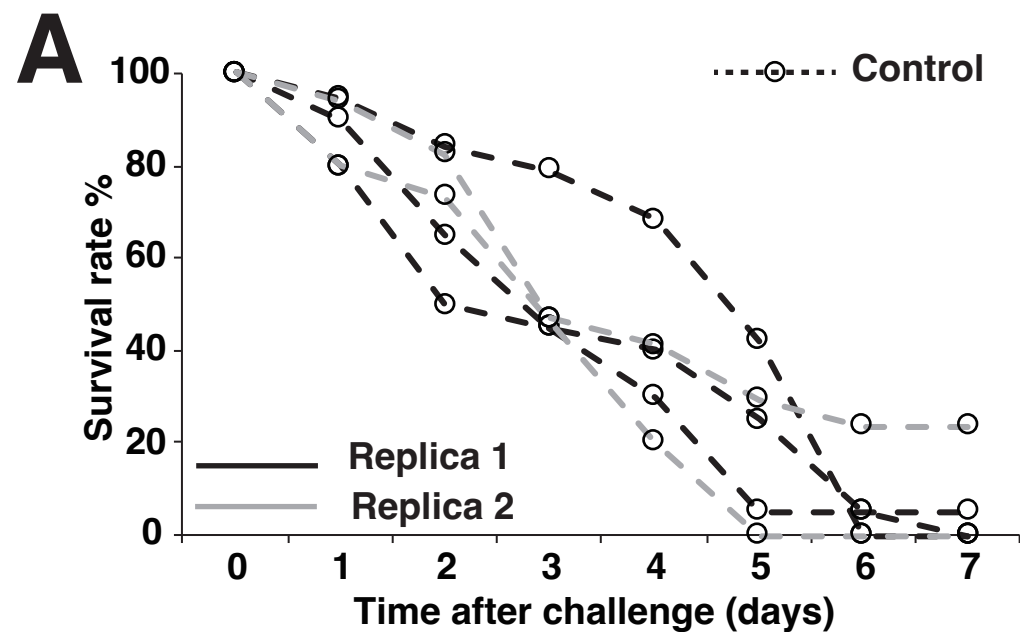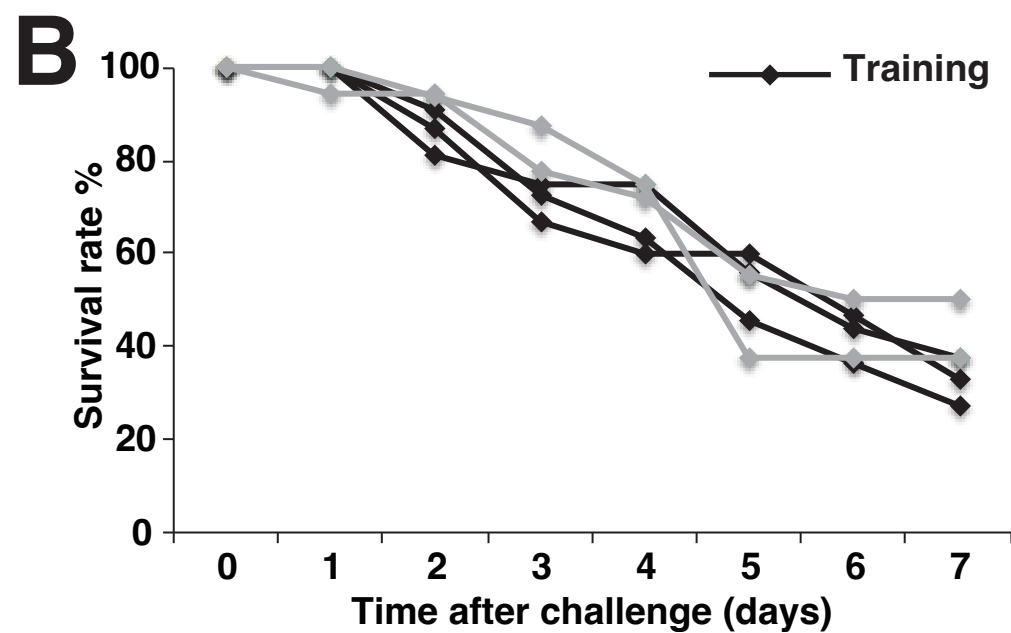

# Fig. S2

## A

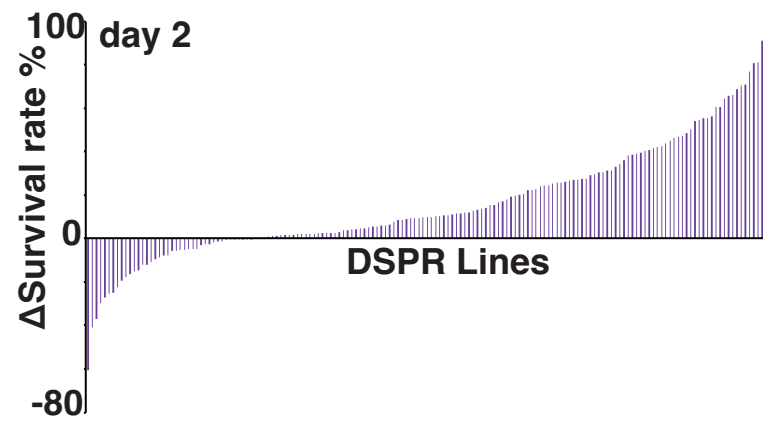

## B

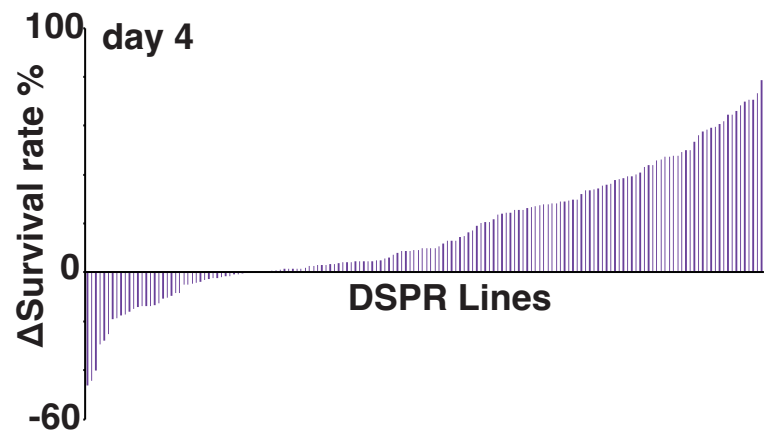

## C

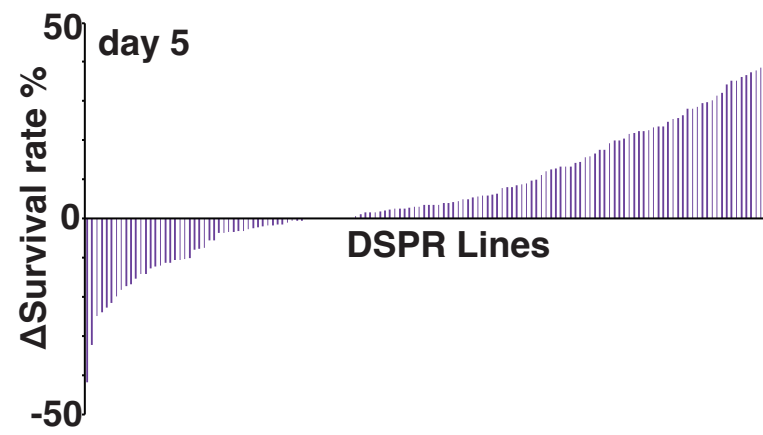

## D

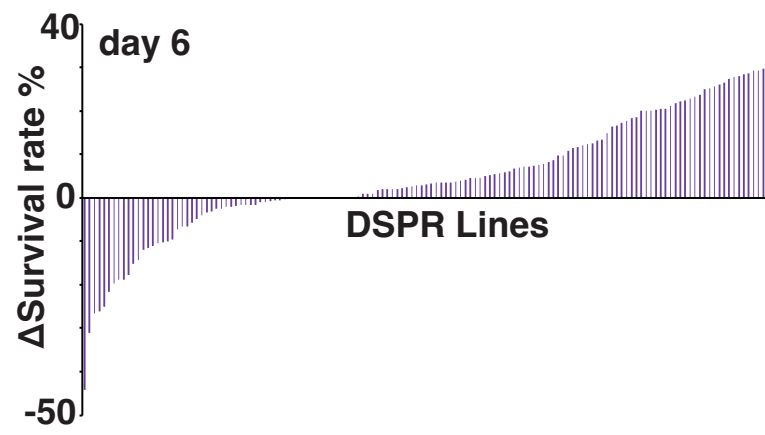

Fig. S3

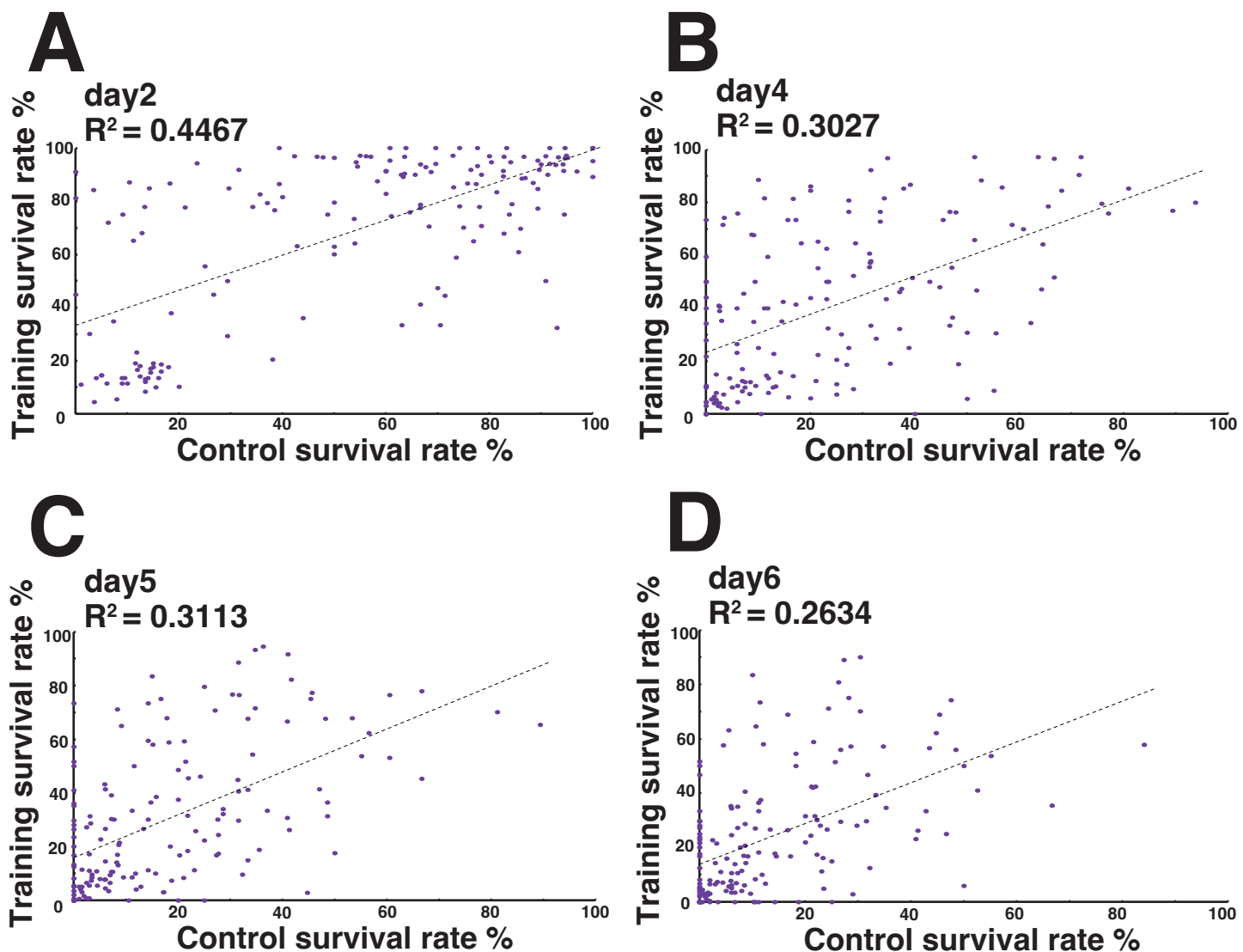

# Fig. S4

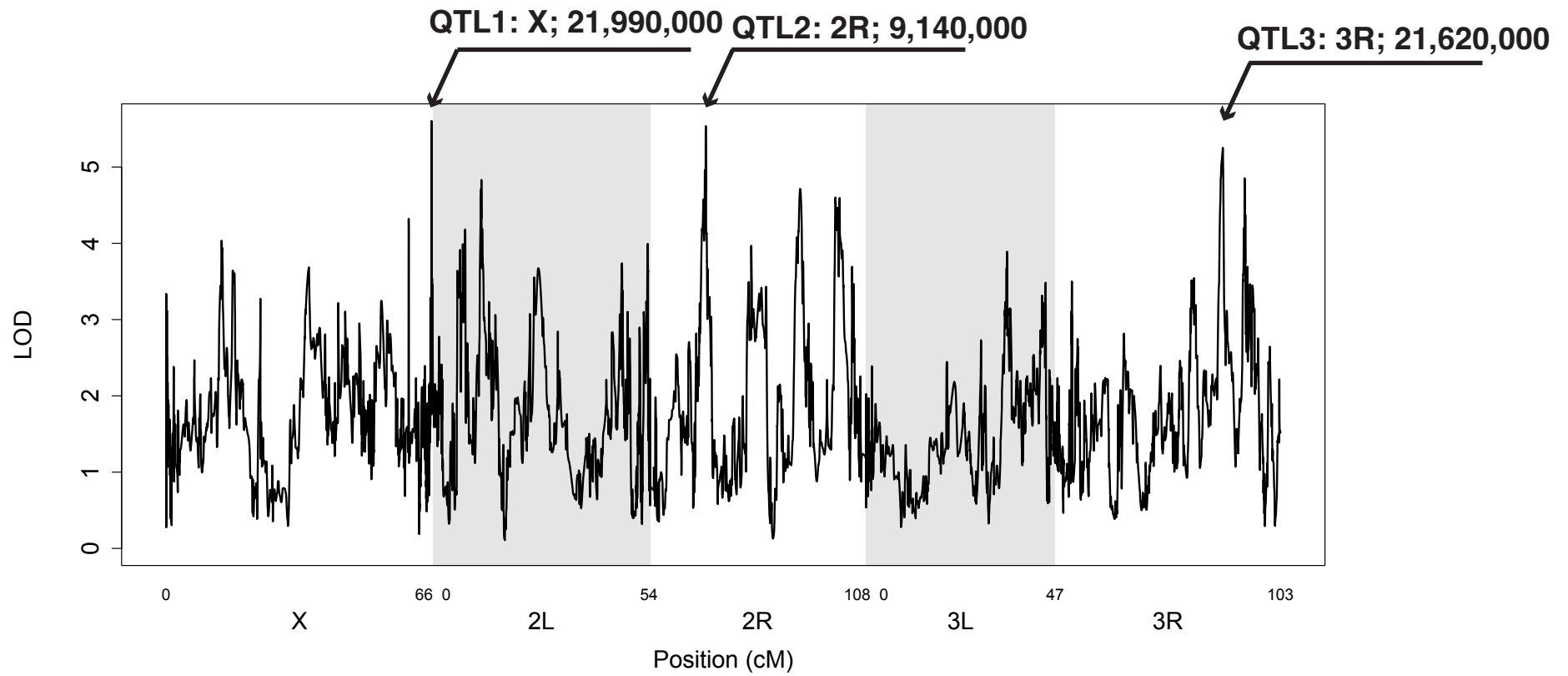

# Fig. S5

## A Peak B1 on day4

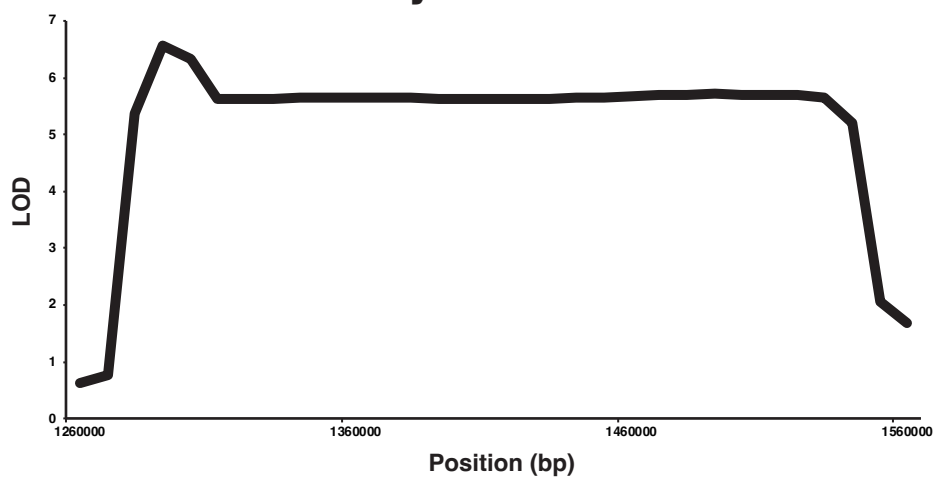

## B Peak B1 on day6

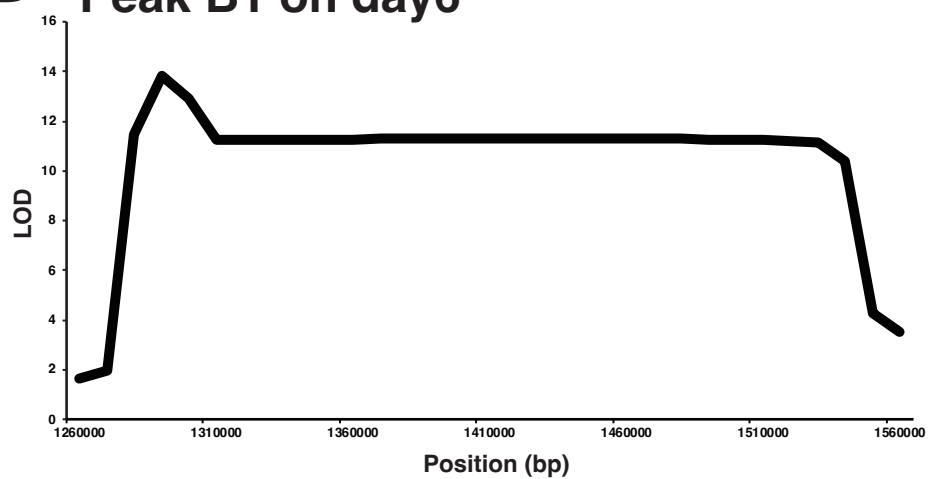

# Fig. S6

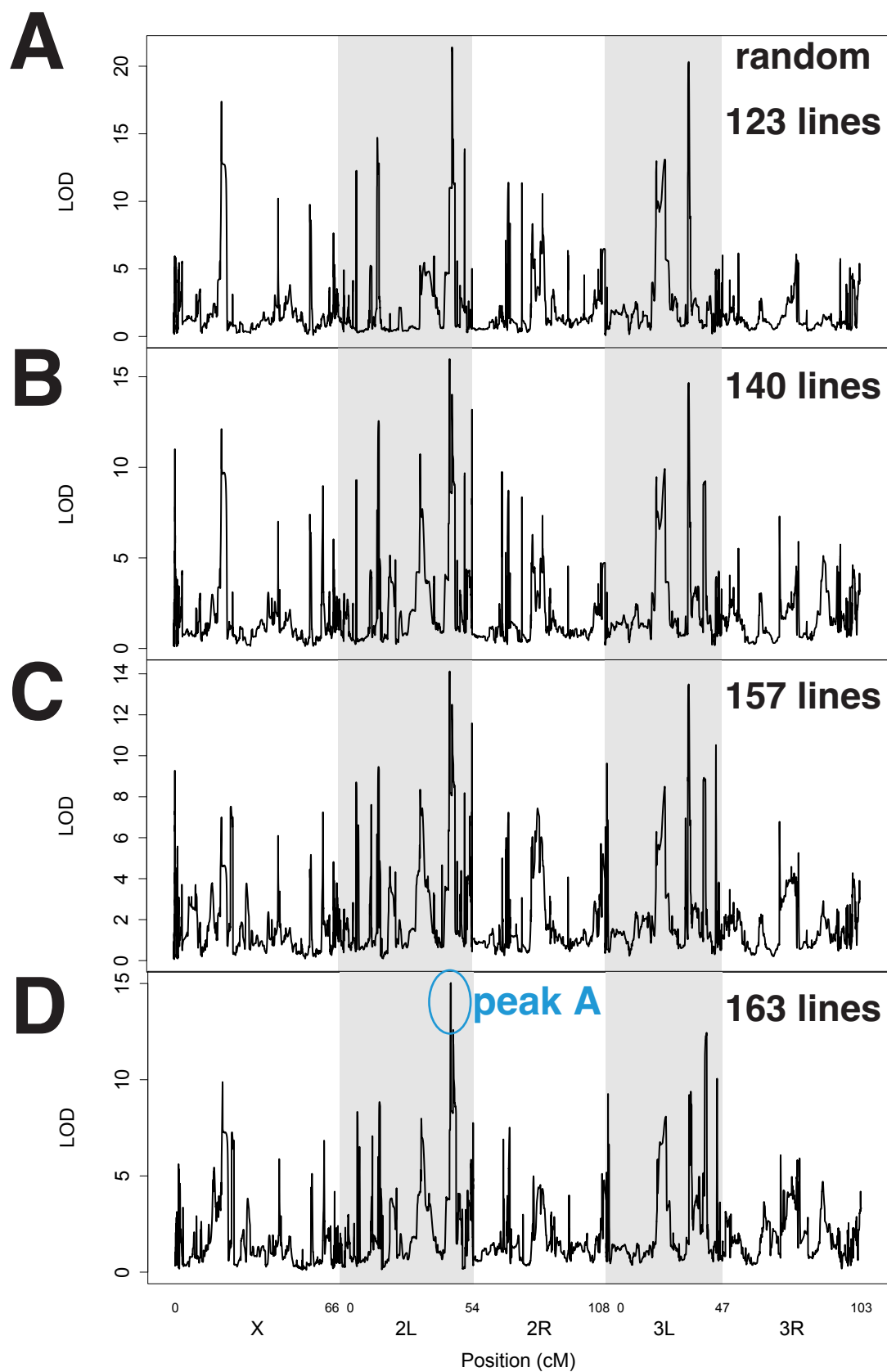

Fig. S7

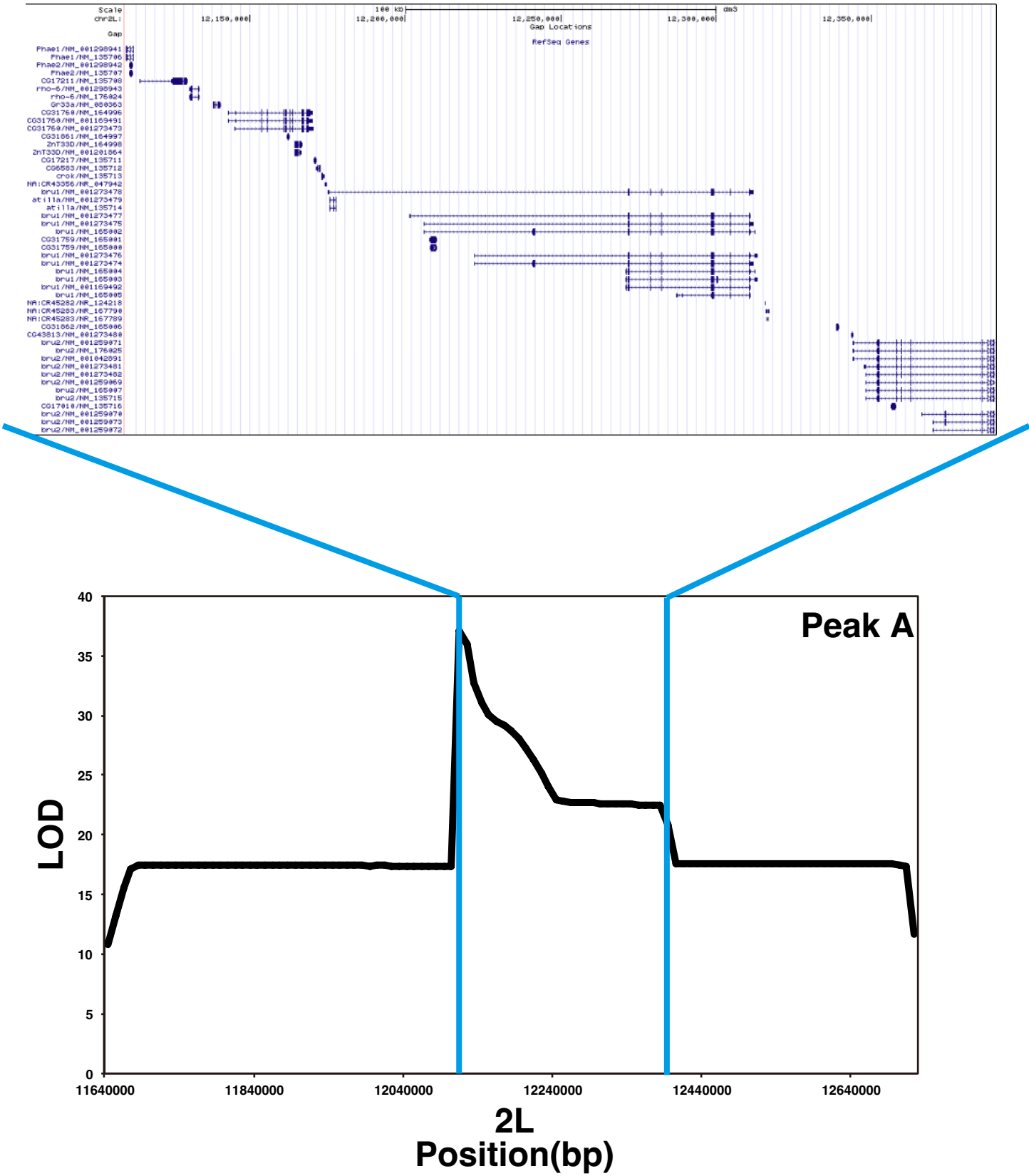

Fig. S8

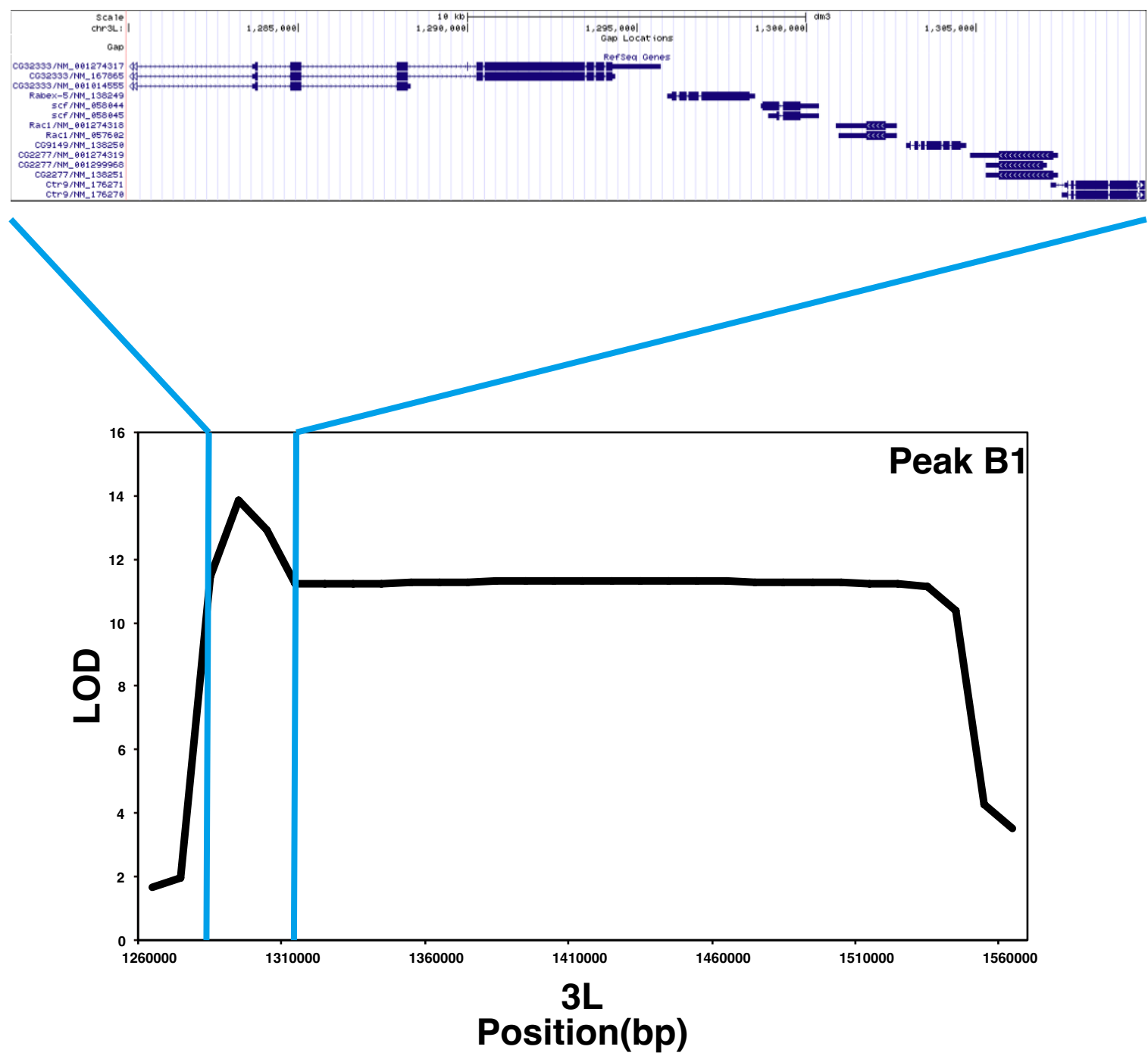

Fig. S9

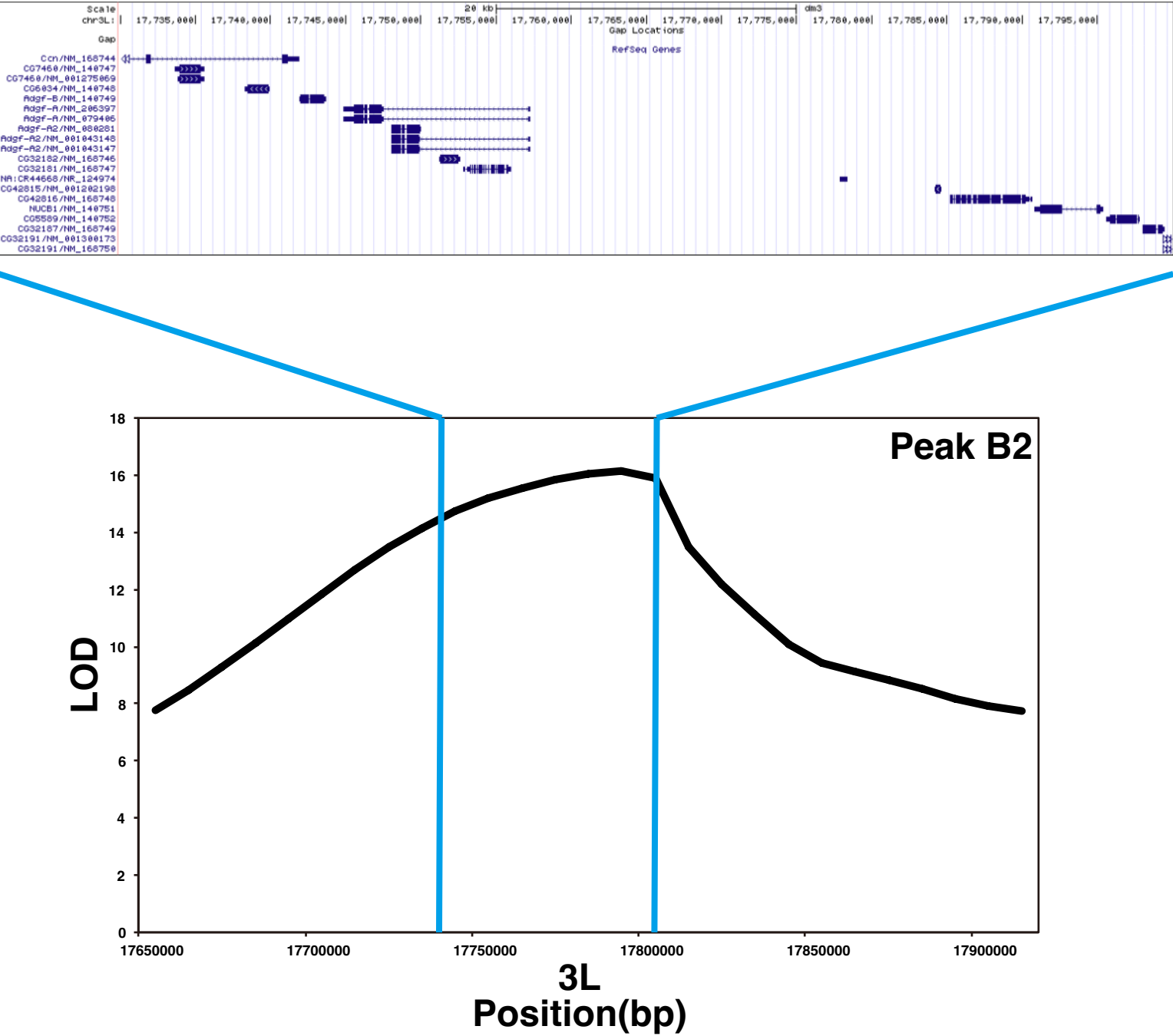

[illegible]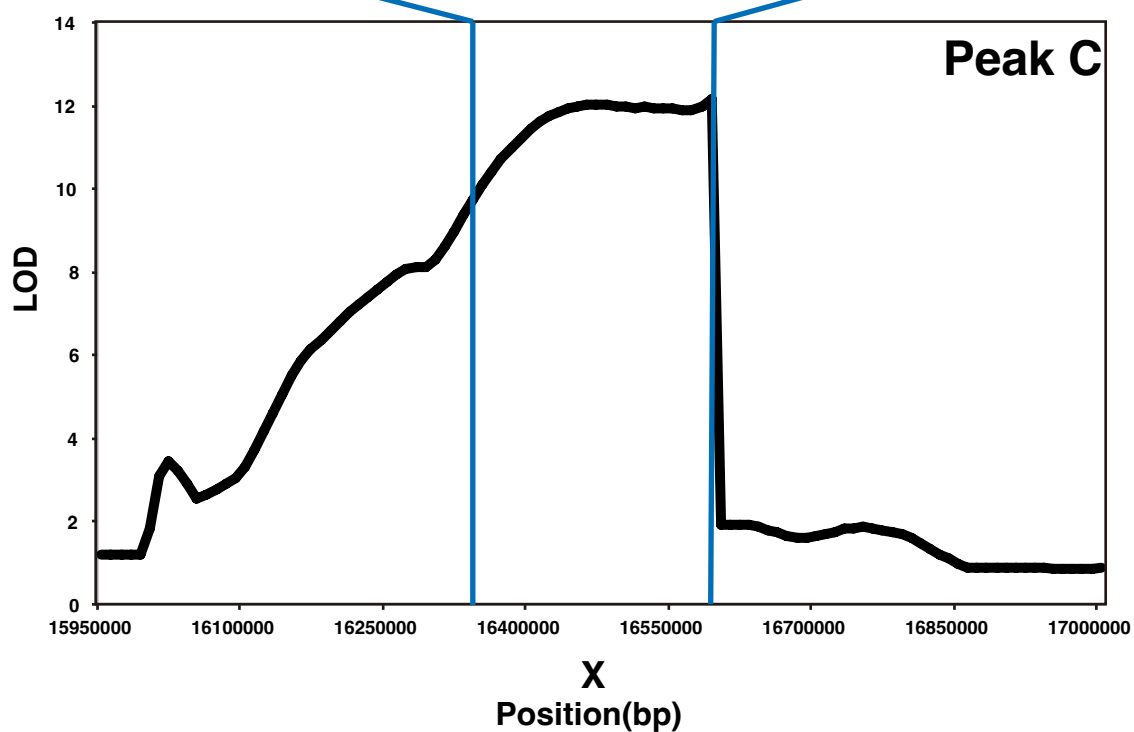

Fig. S11

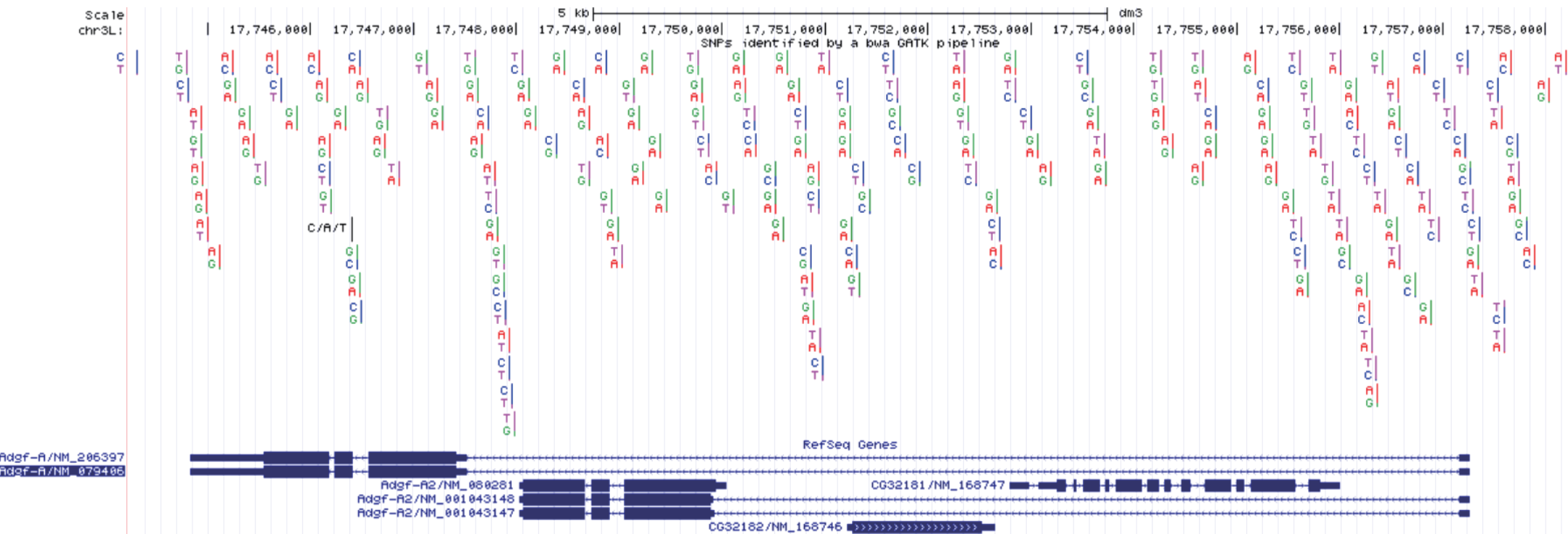

Supplement: Supplementary Figure 1 — Reproducibility of survival rates in our assay system. (A) Survival rates of wild-type flies (Oregon-R) after Sa challenge under control conditions. (B) Survival rates of Oregon-R after Sa challenge under training conditions. The survival assay was performed in two independent experiments consisting of five biological replicates in total. Differences among five replicates in (A) and (B) were not statistically significant (log-rank test). The numbers of flies used in these experiments were (A) 91 (control) and (B) 76 (training). [file Image_1.pdf]
